# Supplementary material for: An open resource combining multi-contrast MRI and microscopy in the macaque brain
Source: Nat Commun. 2023 Jul 19;14:4320. doi: 10.1038/s41467-023-39916-1 (PMC10356772; doi:10.1038/s41467-023-39916-1)
Supplement: Supplementary file 1 — SupplementaryInformation [file 41467_2023_39916_MOESM1_ESM.pdf]

Supplementary material to support the manuscript "An open resource combining multi-contrast MRI and microscopy in the macaque brain" by Howard AFD *et al.*.

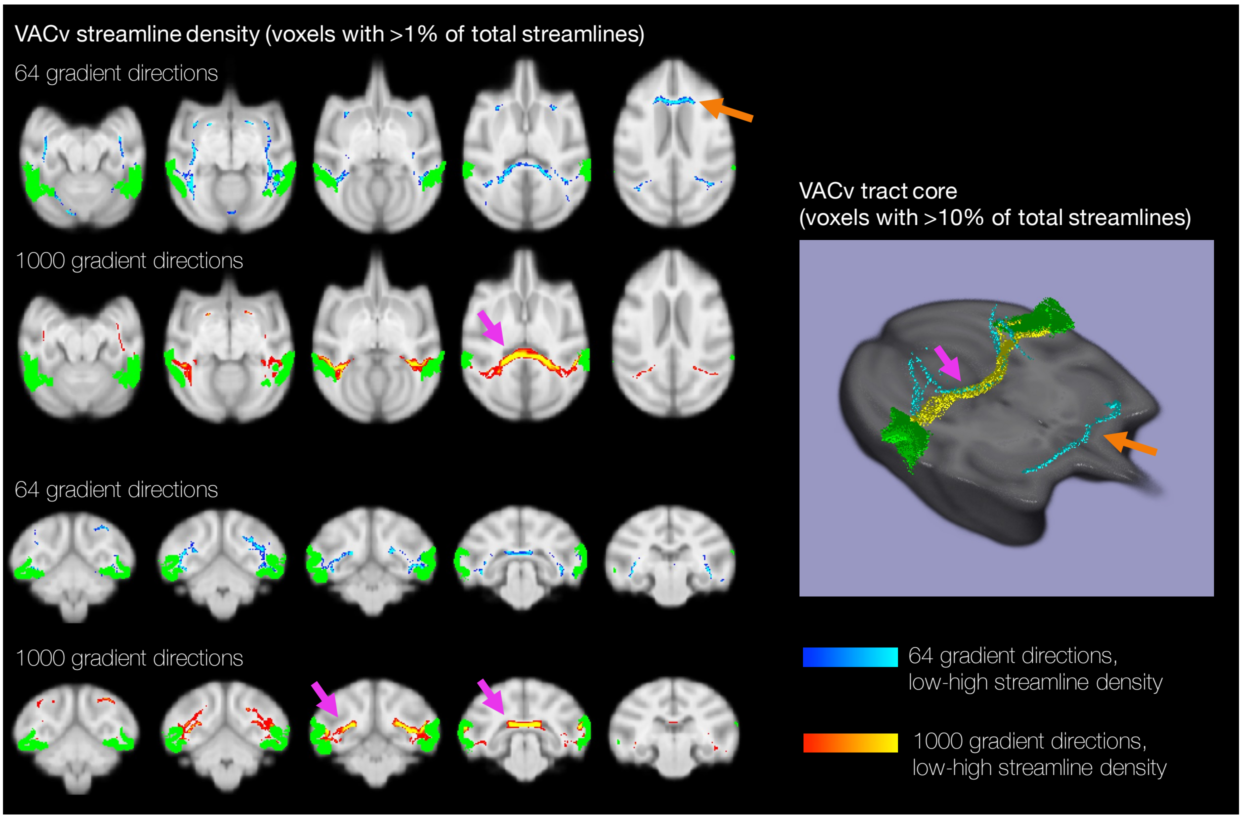

Supplementary Figure 1: Tractography reconstruction of streamlines connecting the left/right anterior visual area (VACv in the RM parcellation, shown in green) from postmortem diffusion MRI ($b = 10\,\mathrm{ms}/\mu\mathrm{m}^2$, 1 mm isotropic) with either 64 or 1000 gradient directions. We observe how the 1000 gradient data supports a large number of streamlines tracking through the corpus callosum (pink arrows), where secondary/tertiary fibre populations in the 1000 gradient data (which are either absent or poorly estimated from the 64 gradient data) facilitate more robust tracking through crossing fibre regions either side of the callosum. In comparison, the 64 direction data includes a high density of streamlines tracking through the anterior commissure (orange arrows), a more convoluted route that may represent a less direct or false positive connection.

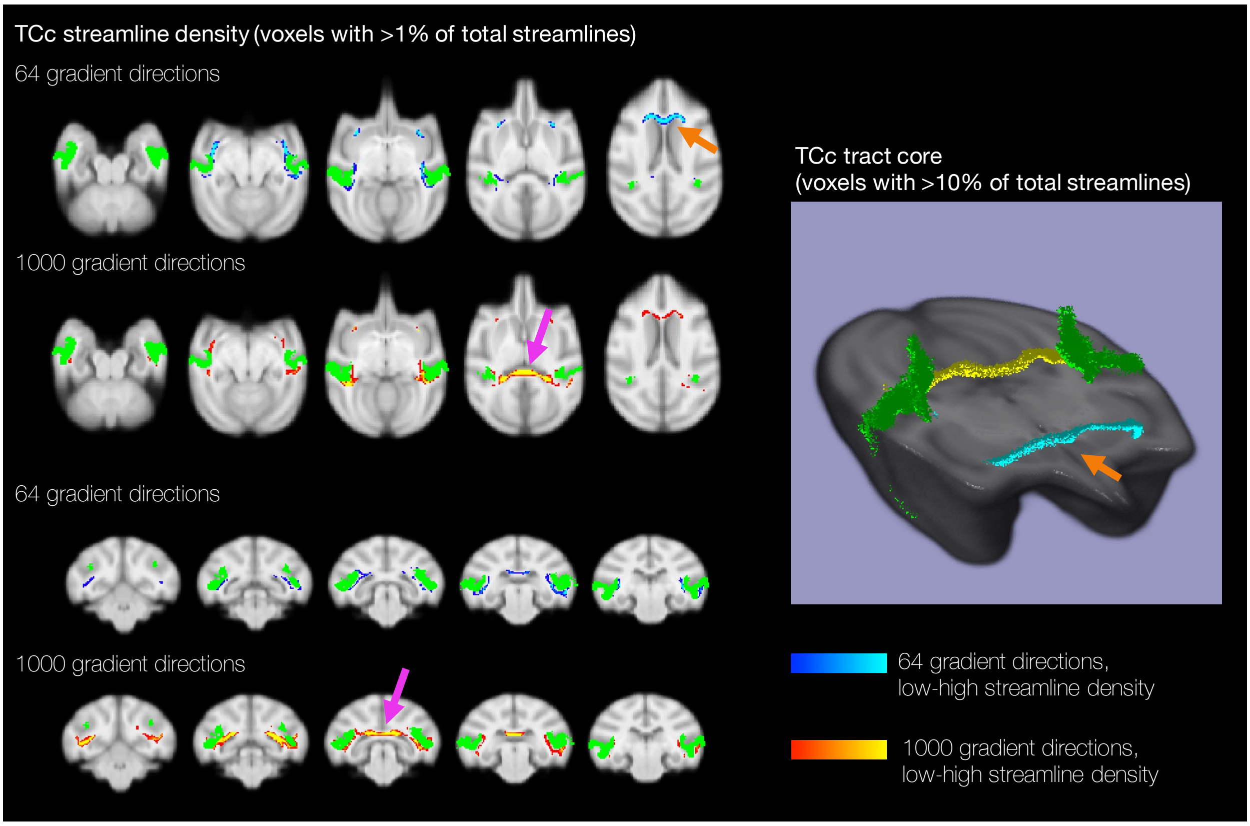

Supplementary Figure 2: Tractography reconstruction of streamlines connecting the left/right central temporal cortex (TCc in the RM parcellation, shown in green) from postmortem diffusion MRI ($b = 10\,\mathrm{ms}/\mathrm{\mu m}^2$, 1 mm isotropic) with either 64 or 1000 gradient directions. We observe similar results to the VACv, with streamlines mostly tracking through the corpus callosum (pink arrows) in the 1000 gradient data, and a secondary pathway through the anterior commissure (orange arrows) in the 64 gradient data.

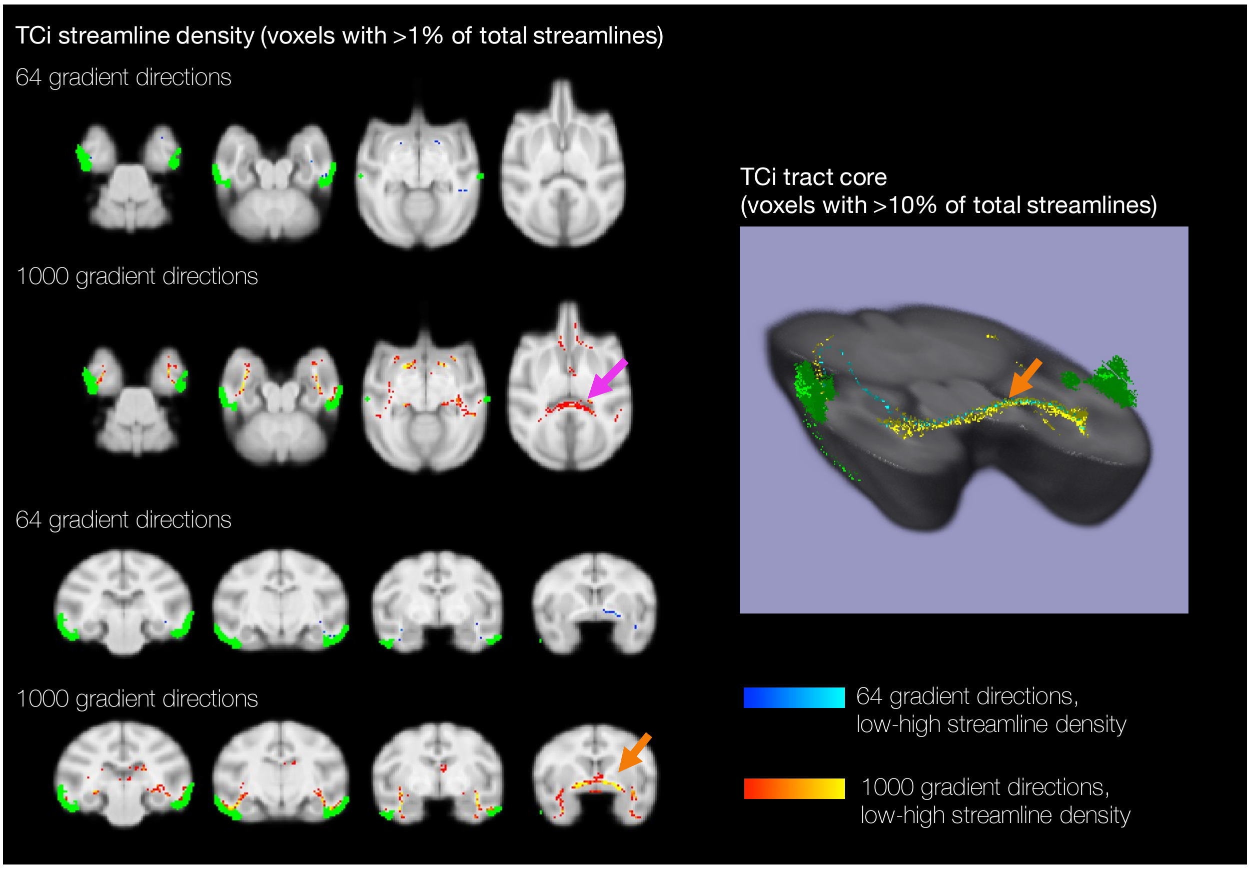

Supplementary Figure 3: Tractography reconstruction of streamlines connecting the left/right inferior temporal cortex (TCi in the RM parcellation, shown in green) from postmortem diffusion MRI ($b = 10\,\text{ms}/\mu\text{m}^2$, 1 mm isotropic) with either 64 or 1000 gradient directions. Both the 64 and 1000 gradient data support a tract core crossing the anterior commisure (orange arrows). The 1000 gradient data also supports some streamlines crossing the callosum (pink arrow). These results are less robust due to the relatively low number of streamlines successfully tracking between the left/right regions of interest (< 10 valid streamlines in the 64 gradient data, and a few hundred in the 1000 gradient data), compared to tens of thousands of streamlines in the 1000 gradient direction TCc and VACv results.

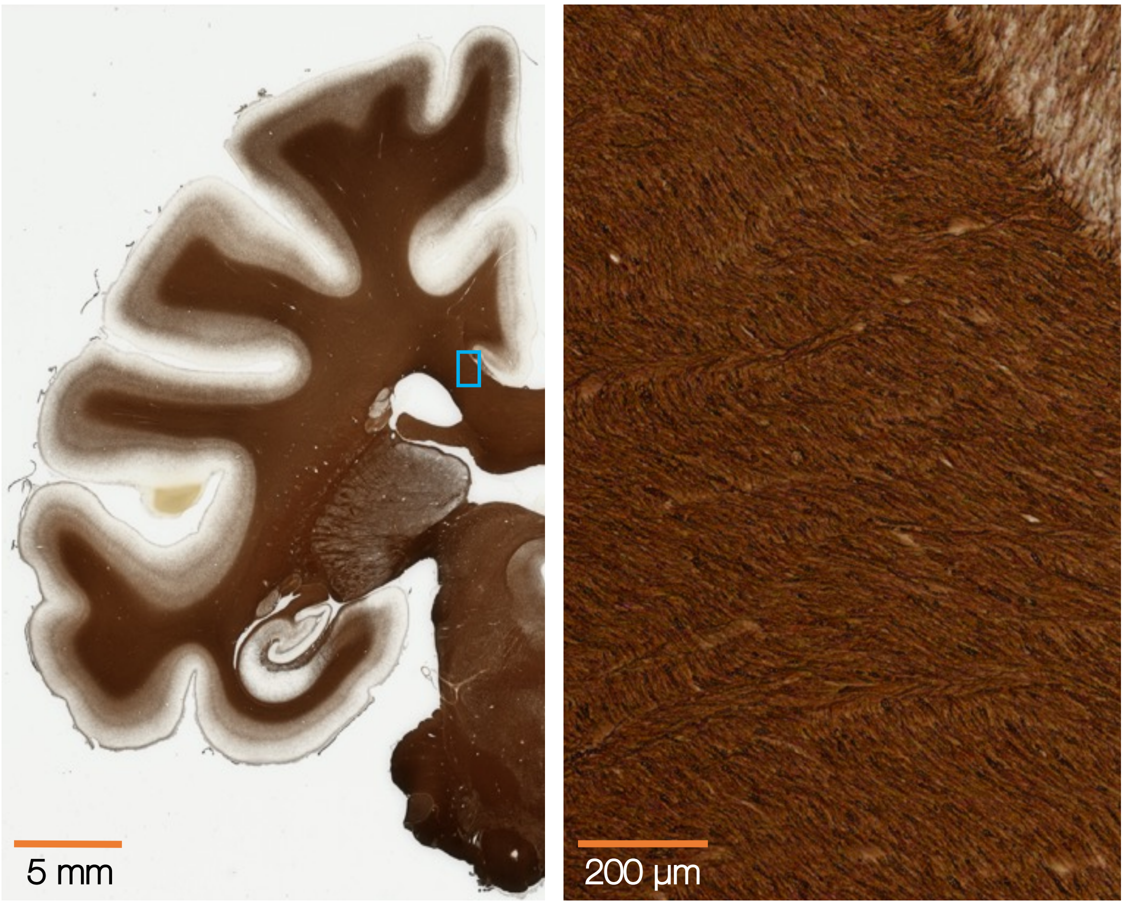

Supplementary Figure 4: An enlarged version of the undulating fibres in the corpus callosum, as described in Figure 4.

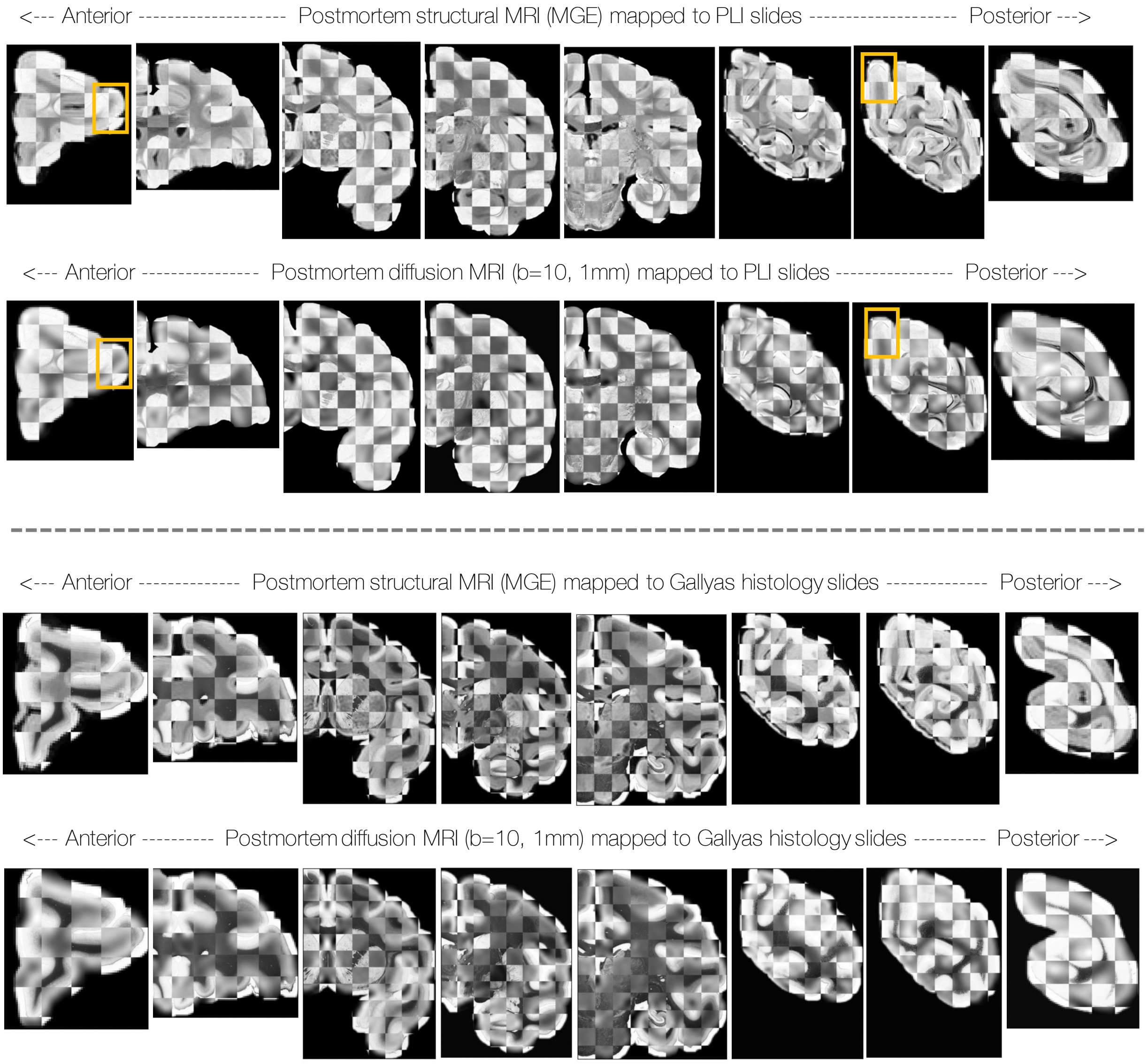

Supplementary Figure 5: Example registration outputs when mapping either the postmortem structural MRI (MGE, 0.3 mm isotropic) or postmortem diffusion MRI ($b = 10\,\mathrm{ms}/\mu\mathrm{m}^2$, 1 mm isotropic) data onto the microscopy plane. Each box in the mosaic image alternates between showing the co-registered MRI output (lower resolution, black background at tissue edges) or the native microscopy image (higher resolution, white background at tissue edge). 1 in 20 slides are shown for PLI (top, inclination image) and Gallyas histology (bottom, RGB structure tensor output converted to greyscale). Note the diffusion images are blurred due to interpolation. The yellow boxes indicate regions in which the registration is less accurate. In the interest of space, only the right hemisphere is shown.

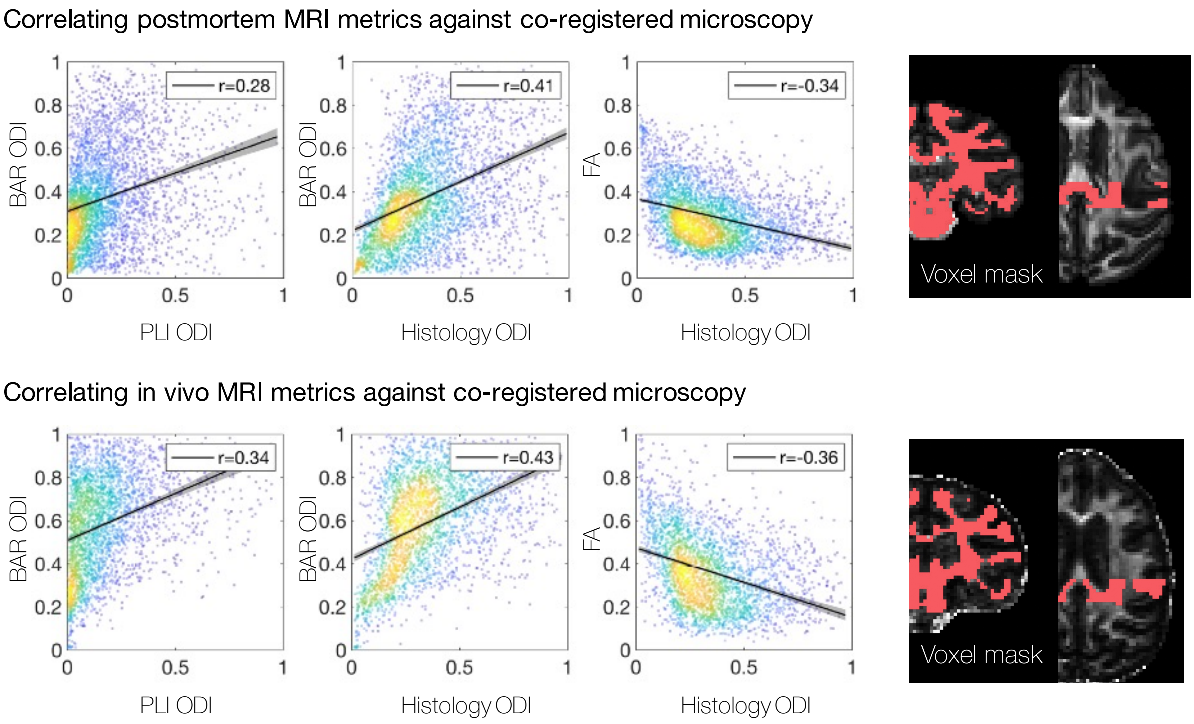

Supplementary Figure 6: Estimates of the Ball and Rackets orientation dispersion index (BAR ODI) and DTI fractional anisotropy (FA) from both postmortem (top) and in vivo (bottom) diffusion MRI are correlated against estimates of ODI from both PLI and myelin histology. The postmortem BAR ODI and FA were estimated from $b = 10\,\mathrm{ms/\mu m^2}$, 1 mm data, and the in vivo MRI metrics from $b = 1\,\mathrm{ms/\mu m^2}$, 1 mm data. Similar relationships were found in vivo and postmortem, though with higher estimates of BAR ODI in vivo. Each point represents a single voxel from the mask shown on the right with a "slab" in MRI space representing 20 consecutive PLI and histology slides in the right hemisphere ($\sim$ 3700 voxels). The blue-yellow colours indicate a low-high density of points. The black lines show the line of best fit, the grey lines indicate the 95% confidence intervals, and r is the correlation coefficient.

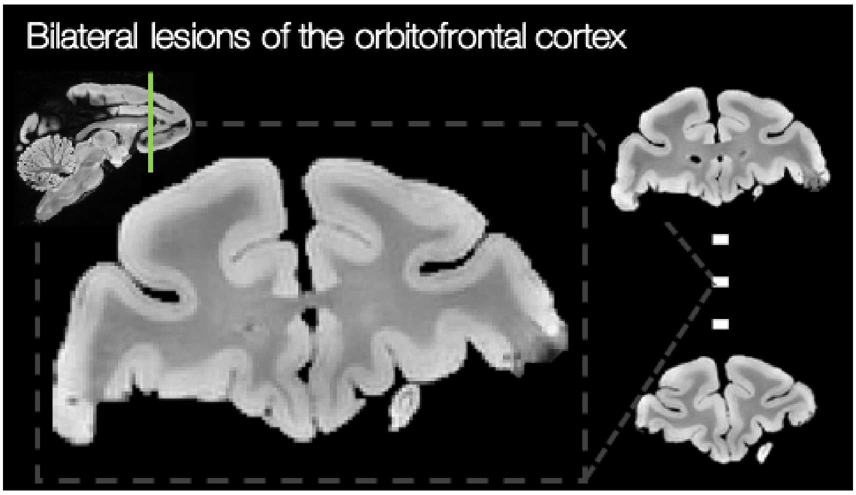

Supplementary Figure 7: Bilateral lesioning of the orbitofrontal cortex where postmortem structural MR images show the extent of the lesion. The top image is the most posterior, and the bottom the most anterior, of those shown. The lesion occurred approximately 1 year prior to the scans shown.

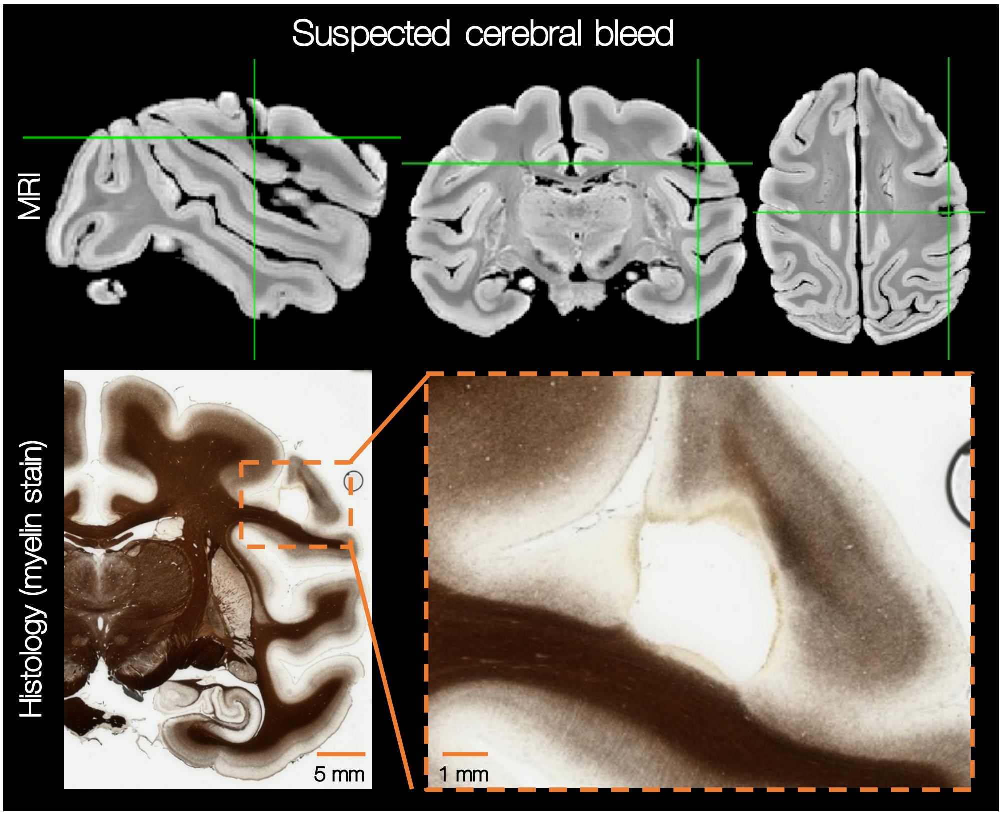

Supplementary Figure 8: Both the postmortem structural MRI (top) and myelin-stained histology data (bottom) show a region of tissue damage in the left hemisphere of the BigMac brain. The region of damaged tissue can be tracked through 20 myelin-stained slides and this region is also visible in the most recent in vivo MRI. The tissue damage may be indicative of a postoperative cerebral bleed, though there was no indication of this at the time.

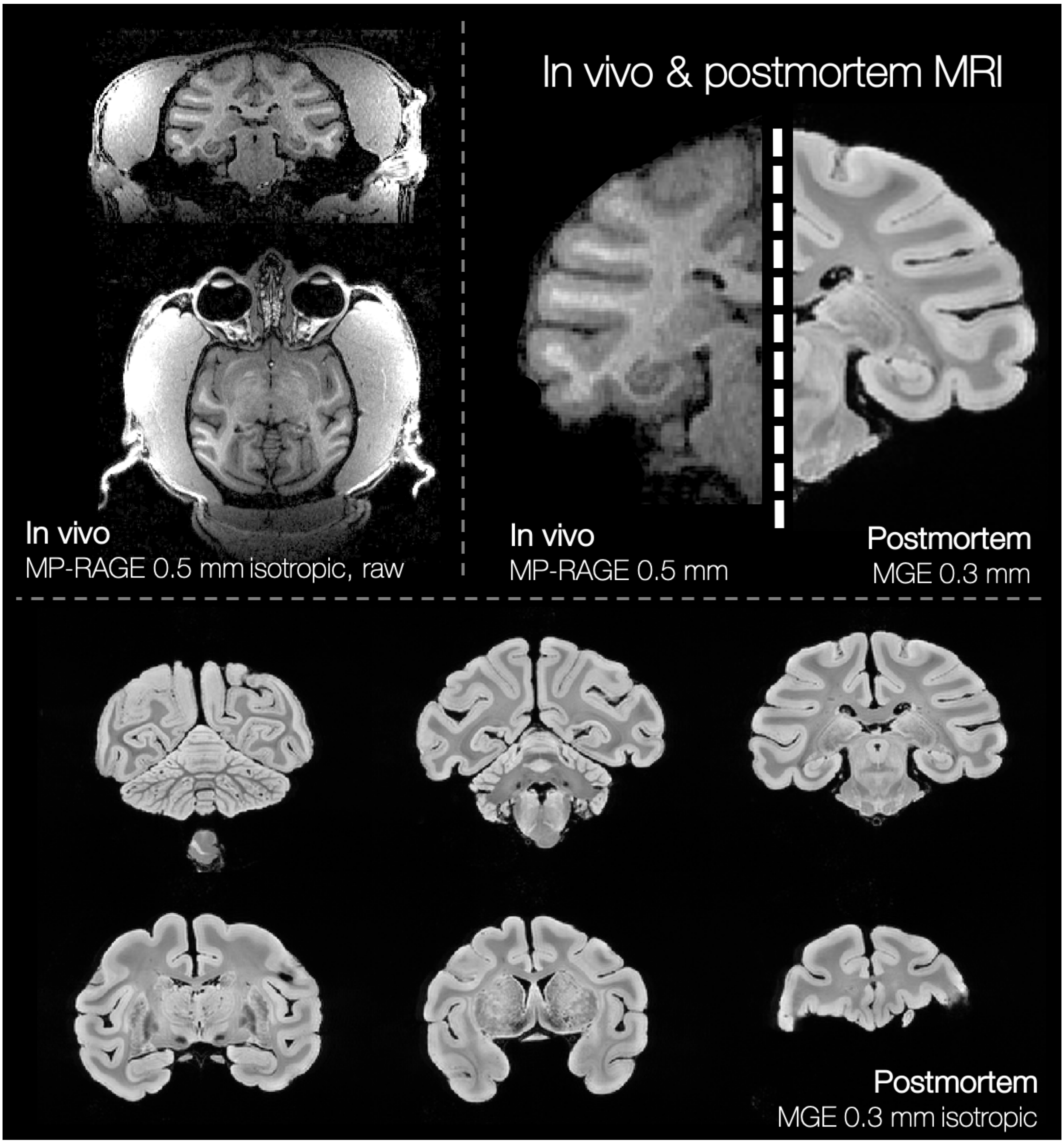

Supplementary Figure 9: The BigMac dataset includes both in vivo (structural, functional and diffusion MRI) and postmortem (structural MRI, diffusion MRI and microscopy) data in the same animal. Here we compare example structural images from both the in vivo and postmortem protocol. Though there is inverted contrast between the in vivo and postmortem structural images, the data is highly complementary. Furthermore, in the high-quality postmortem data (bottom) we can see the anatomy in detail due to the high 0.3 mm isotropic resolution. The top left image is the most posterior and the bottom right the most anterior, where we again see the bilateral lesion of the orbitofrontal cortex.

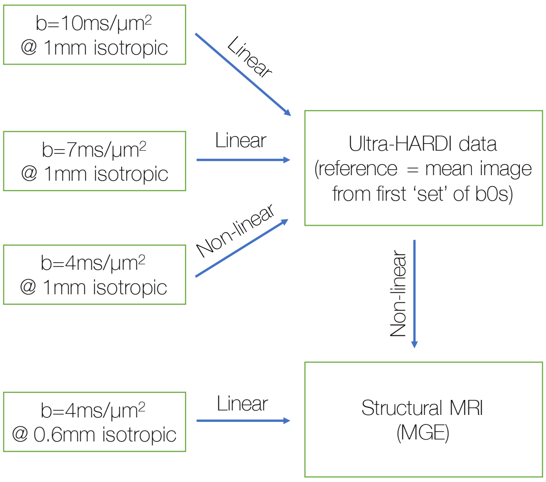

Supplementary Figure 10: Co-registration of the BigMac postmortem MRI data.

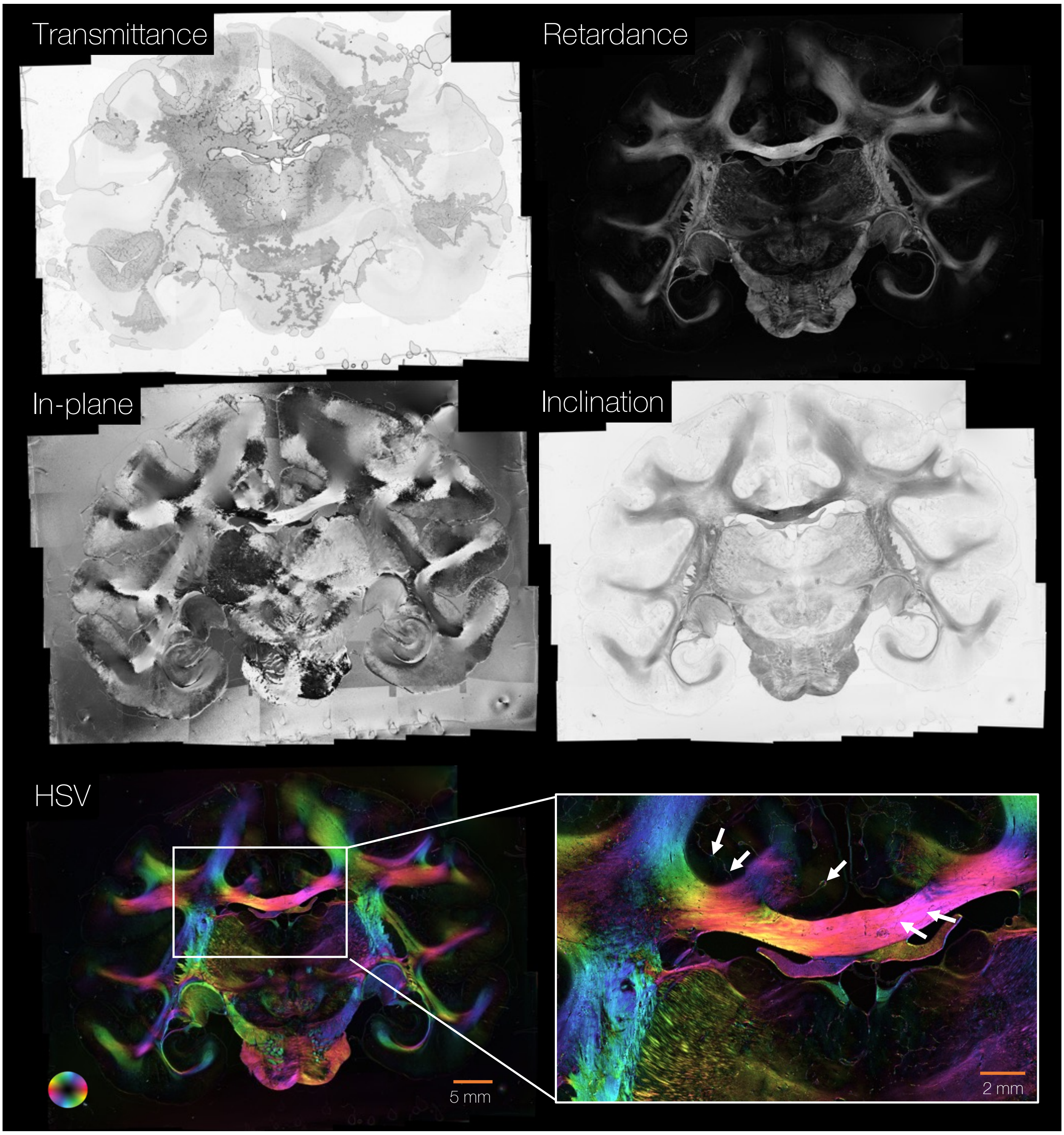

Supplementary Figure 11: Example transmittance, retardance, in-plane and inclination maps from PLI. The in-plane and retardance images are combined in a composite HSV image. Though the slide is badly affected by bubbles and artefacts (see transmittance image), the artefacts appear to have little effect on the other PLI maps (retardance, in-plane and inclination). Consequently, only very minor artefacts are seen in the HSV image (white arrows). Whole slide PLI were acquired for 192 sections throughout the BigMac brain: 176 in the anterior half and 76 in the posterior.

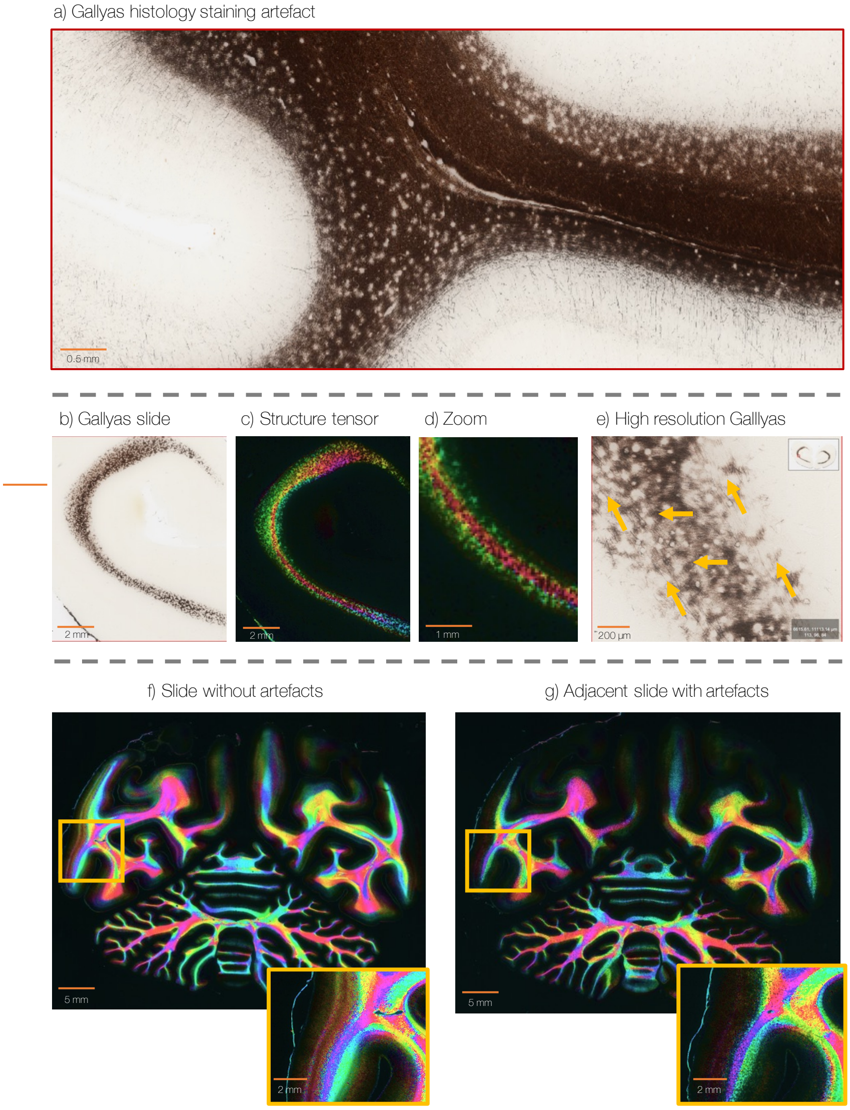

Supplementary Figure 12: Artefacts seen in the posterior Gallyas-stained slides. a) The myelin stain appears patchy in the white matter with relatively little stain density in the grey matter. b-e) Structure tensor analysis of the slides with the staining artefact produces remarkably reasonable fibre orientations, particularly in the white matter. The orientations are smoothly varying and we are able to delineate the presence of multiple fibre bundles. f-g) When comparing structure tensor analysis of adjacent slides, one with and the other without the artefact, we see very similar orientations within the white matter, but reduced grey matter contrast in the slide with staining artefact. Similar artefacts were found in many of the 77 posterior myelin-stained slides.
