# Supplementary material for: An open resource combining multi-contrast MRI and microscopy in the macaque brain
Source: Nat Commun. 2023 Jul 19;14:4320. doi: 10.1038/s41467-023-39916-1 (PMC10356772; doi:10.1038/s41467-023-39916-1)
Supplement: Supplementary file 3 — Reporting Summary [file 41467_2023_39916_MOESM3_ESM.pdf]

nature portfolio

Corresponding author(s): Amy Howard

Last updated by author(s): 2023/05/10

Reporting Summary

Nature Portfolio wishes to improve the reproducibility of the work that we publish. This form provides structure for consistency and transparency in reporting. For further information on Nature Portfolio policies, see our Editorial Policies and the Editorial Policy Checklist.

Statistics

For all statistical analyses, confirm that the following items are present in the figure legend, table legend, main text, or Methods section.

n/a	Confirmed	
☐	☒	The exact sample size (*n*) for each experimental group/condition, given as a discrete number and unit of measurement
☒	☐	A statement on whether measurements were taken from distinct samples or whether the same sample was measured repeatedly
☐	☒	The statistical test(s) used AND whether they are one- or two-sided<br>*Only common tests should be described solely by name; describe more complex techniques in the Methods section.*
☒	☐	A description of all covariates tested
☒	☐	A description of any assumptions or corrections, such as tests of normality and adjustment for multiple comparisons
☐	☒	A full description of the statistical parameters including central tendency (e.g. means) or other basic estimates (e.g. regression coefficient) AND variation (e.g. standard deviation) or associated estimates of uncertainty (e.g. confidence intervals)
☐	☒	For null hypothesis testing, the test statistic (e.g. *F*, *t*, *r*) with confidence intervals, effect sizes, degrees of freedom and *P* value noted<br>*Give P values as exact values whenever suitable.*
☒	☐	For Bayesian analysis, information on the choice of priors and Markov chain Monte Carlo settings
☒	☐	For hierarchical and complex designs, identification of the appropriate level for tests and full reporting of outcomes
☐	☒	Estimates of effect sizes (e.g. Cohen's *d*, Pearson's *r*), indicating how they were calculated

Our web collection on statistics for biologists contains articles on many of the points above.

Software and code

Policy information about availability of computer code

Data collection	All MRI data was acquired using standard Bruker sequences. Polarised light image were acquired on a Leica microscope (DM4000 B) using Leica Application Suite X software (LAS V4.12). Histology slides were digitised on an Aperio ScanScope Turbo AT slidescanner (Leica) with console software V102.0.0.74.
Data analysis	MRI data were analysed using the FMRIB Software Library (FSL 6.0.5/6.0.6) which is openly available via https://fsl.fmrib.ox.ac.uk/fsl/fslwiki using scripts available via https://git.fmrib.ox.ac.uk/amyh/bigmacanalysis. Mrtrix3 (V3.12/3.15) was used for Gibbs ringing correction: https://www.mrtrix.org/download/ and https://github.com/jdtournier/mrdegibbs3D. In vivo MRI were preprocessed using the MR comparative anatomy toolbox (MrCat) which is available from https://github.com/neuroecology/MrCat. T1maps were generated using qMRLab https://qmrlab.readthedocs.io/en/master/ (V2.4.1) and MATLAB 2021a. Diffusion data with multiple tensor encoding were analysed using the multi-dimensional diffusion MRI toolbox, https://github.com/markus-nilsson/md-dmri and MATLAB 2021a.<br><br>PLI processing (to generate the in-plane, retardance, transmittance and inclination maps), and structure tensor analysis of histological slides, were performed using in house MATLAB scripts (MATLAB 2018-2021a). MRI-microscopy co-registration was performed using TIRL V2.1.3b1 which is openly available as part of FSL and available via git at https://git.fmrib.ox.ac.uk/ihuszar/tirl.<br><br>Code to recreate the analyses performed in the paper (Figures 3, 5 and 6 and Supplementary Figures 1, 2, 3, 5 & 6) are openly available via https://git.fmrib.ox.ac.uk/amyh/bigmacanalysis. At the current time, we do not provide code to directly recreate the outputs in Figure 7 (hybrid tractography) as this is the subject of active, ongoing work within the group. Our code, alongside tutorials on how to compare MRI and microscopy in BigMac, are available via the online documentation: https://open.win.ox.ac.uk/pages/amyh/bigmacdocumentation/index.html.

All python/MATLAB scripts were run using python 3 or MATLAB 2021a.

For manuscripts utilizing custom algorithms or software that are central to the research but not yet described in published literature, software must be made available to editors and reviewers. We strongly encourage code deposition in a community repository (e.g. GitHub). See the Nature Portfolio guidelines for submitting code & software for further information.

Data

Policy information about availability of data

All manuscripts must include a data availability statement. This statement should provide the following information, where applicable:

- Accession codes, unique identifiers, or web links for publicly available datasets
- A description of any restrictions on data availability
- For clinical datasets or third party data, please ensure that the statement adheres to our policy

The BigMac data (including minimally preprocessed data) are openly available via the Digital Brain Bank: https://open.win.ox.ac.uk/DigitalBrainBank/#/datasets/anatomist. Example data can be accessed via the online viewer and the full dataset is available via a material transfer agreement to ensure the data is used for purposes which satisfy research ethics and funding requirements. Further documentation (including example images of all modalities) is available via https://open.win.ox.ac.uk/pages/amyh/bigmacdocumentation/index.html. Since the full dataset requires a considerable amount of memory (~ 1.8 TB), users may wish to request only a subset of relevant data. To facilitate this, the documentation includes an extensive file tree, listing available files as well as approximate memory requirements for different parts of the data. Additional data linking behaviour to fMRI (z-statistics maps) can be accessed via Grohn 2020 (doi:10.1371/journal.pbio.3000899) and Sallet 2020 (doi:10.1371/journal.pbio.3000605), and similar in vivo MRI (structural and resting-state fMRI) are available for another 19 subjects for cross-subject comparisons via the primate data exchange (PRIME-DE): https://fcon_1000.projects.nitrc.org/indi/PRIME/oxford.html. Source data are provided with this paper.

Human research participants

Policy information about studies involving human research participants and Sex and Gender in Research.

Reporting on sex and gender	No human research participants were analysed.
Population characteristics	See above.
Recruitment	See above.
Ethics oversight	See above.

Note that full information on the approval of the study protocol must also be provided in the manuscript.

Field-specific reporting

Please select the one below that is the best fit for your research. If you are not sure, read the appropriate sections before making your selection.

☒ Life sciences ☐ Behavioural & social sciences ☐ Ecological, evolutionary & environmental sciences

For a reference copy of the document with all sections, see nature.com/documents/nr-reporting-summary-flat.pdf

Life sciences study design

All studies must disclose on these points even when the disclosure is negative.

Sample size	A single brain was studied since data acquisition is very time intensive.
Data exclusions	No excluded data.
Replication	Analyses were performed on a single brain and are yet to be replicated elsewhere. This is due to the very time intensive nature of data acquisition and curation which takes several years for a single brain. Acquiring similar data on additional brains is the subject of ongoing and future work.
Randomization	No groups were defined.
Blinding	Blinding was not relevant as we did not perform any statistical tests (bar correlation) or make comparisons between groups.

Reporting for specific materials, systems and methods

We require information from authors about some types of materials, experimental systems and methods used in many studies. Here, indicate whether each material, system or method listed is relevant to your study. If you are not sure if a list item applies to your research, read the appropriate section before selecting a response.

Materials & experimental systems

n/a	Involved in the study
☒	☐ Antibodies
☒	☐ Eukaryotic cell lines
☒	☐ Palaeontology and archaeology
☐	☒ Animals and other organisms
☒	☐ Clinical data
☒	☐ Dual use research of concern

Methods

n/a	Involved in the study
☒	☐ ChIP-seq
☒	☐ Flow cytometry
☐	☒ MRI-based neuroimaging

Animals and other research organisms

Policy information about studies involving animals; ARRIVE guidelines recommended for reporting animal research, and Sex and Gender in Research

Laboratory animals	Rhesus Macaque (Macaca mulatta). In vivo data were acquired at 4 and 10.7 years. Postmortem data was acquired at 11.7 years of age.
Wild animals	No wild animals were involved.
Reporting on sex	The animal was male, but sex was not considered during the analysis.
Field-collected samples	This study did not involve animals from the field.
Ethics oversight	In vivo, the animal was cared for, and data were acquired by researchers at the University of Oxford, UK. All procedures were performed under licenses from the United Kingdom (UK) Home Office in accordance with the UK Animals (Scientific Procedures) Act 1986 and with European Union guidelines (EU Directive 2010/63/EU).<br>No licenses were required for postmortem data collection.

Note that full information on the approval of the study protocol must also be provided in the manuscript.

Magnetic resonance imaging

Experimental design

Design type	Resting-state fMRI
Design specifications	Resting-state data was acquired for 52 min 26 s in one scan session and 26 min 13 s in the second scan session.
Behavioral performance measures	Not acquired as part of this study.

Acquisition

Imaging type(s)	Resting-state fMRI, diffusion MRI, structural MRI, T1 maps (inversion recovery)
Field strength	3T in vivo, 7T postmortem
Sequence & imaging parameters	In vivo MRI:<br>Structural MRI images were acquired using a T1-weighted Magnetization Prepared - RApid Gradient Echo (MP-RAGE) sequence with 0.5 mm isotropic resolution, TE/TR = 4.01 ms/2.5 s and 128 slices. Whole brain fMRI data (BOLD) were acquired with echo planar imaging (EPI) and 2 mm isotropic resolution: TE/TR = 19 ms/2 s, 1600 volumes for Session 1 and 800 volumes for Session 2. This corresponds to 52 min 26 s and 26 min 13 s of data respectively. Diffusion MRI data were acquired using EPI with 1 mm isotropic resolution, TE/TR = 100 ms/8.2 s and a b-value of 1ms/µm2. 1100 diffusion weighted (81 unique gradient directions) and 144 non-diffusion weighted volumes were acquired with both +/- phase encoding directions for Session 1, and 361 diffusion weighted (61 unique gradient directions) and 38 non-diffusion weighted volumes were acquired with both +/- phase encoding directions for Session 2.<br><br>Postmortem MRI:<br>Two structural images were acquired - one with multi gradient echo (MGE 3D) sequence, and one using balanced steady-state free procession (bSSFP). The MGE parameters were: TE/TR = 7.8/97.7 ms, flip angle = 30, 0.3 mm isotropic

resolution, FOV = 76.8 x 76.8 x 76.8 mm. The bSSFP data were acquired using a TRUFI sequence with 16 frequency increments: TE/TR = 3.05/6.1 ms, flip angle = 30, 0.3 mm isotropic resolution, FOV = 76.8 x 76.8 x 76.8 mm. The structural image was formed by averaging the data using root-mean sum of squares.

A T1 map was acquired with an inversion recovery preparation: TE/TR = 8.6 ms/10 s, FOV = 76.8 x 76.8 x 76.8mm, resolution 0.6 mm isotropic and 12 inversion times (TI) from 10 to 6000 ms.

The diffusion-weighted data were acquired using a spin echo multi-slice (DW-SEMS) sequence and single-line readout. The 1mm data acquisition parameters were as follows: TE/TR = 42.4 ms/3.5 s; FOV=76 x 76 x 76 mm; delta/Delta = 14/24 ms; 1 mm isotropic resolution; time per gradient direction = 4.4 mins; b=4ms/μm2 data had G = 12.0 G/cm, 250 gradient directions and 10 non-diffusion weighted volumes; b=7ms/μm2 had G = 15.9 G/cm, 1000 gradient directions and 40 non-diffusion weighted volumes; b=10ms/μm2 had G = 19.1 G/cm, 1000 gradient directions and 40 non-diffusion weighted volumes.

The 0.6 mm b=4 ms/μm2 data followed a different protocol. The acquisition parameters were as follows: TE/TR = 25.4 ms/10 s; FOV=76.8 x 76.8 x 76.8 mm; delta/Delta = 7/13 ms; time per gradient direction = 21.3 mins; b=4 ms/μm2; 0.6 mm isotropic resolution; G = 32 G/cm;128 diffusion-weighted gradient directions; 8 volumes with negligible diffusion weighting.

Data with spherical tensor encoding were also acquired at b-values of 4, 7 and 10 ms/μm2. TE/TR = 42.5 ms/6.4 s; FOV = 76x76x76 mm; 1 mm isotropic resolution. For each b-value, 30 images were acquired with spherical tensor encoding and 1 with negligible diffusion weighting. Complementary data with linear tensor encoding and the same TR were also acquired: delta/Delta = 14/24 ms; 50 gradient directions per shell, plus 2 volumes with negligible diffusion weighting. The gradient amplitude G was adjusted to produced the required b-values of b=4, 7 and 10 ms/μm2.

Area of acquisition: Whole brain

Diffusion MRI: ☒ Used ☐ Not used

Parameters: See above.

Preprocessing

Preprocessing software: FMRIB Software Library (FSL 6.0.5/6.0.6): FLIRT and FNIRT for MRI-MRI registration, BET for brain extraction, FAST for wm/gm tissue segmentation, topup and eddy for susceptibility and eddy current distortion correction of in vivo diffusion MRI, melodic for ICA-based cleaning the resting-state fMRI. MRtrix3: deGibbs3D for 3D Gibbs correction. T1maps were generated using qMRLab https://qmrlab.readthedocs.io/en/master/ (V2.4.1) and MATLAB 2021a.

Normalization: Diffusion MRI data were normalised to the first non-diffusion weighted volume (S0) by fitting a linear trend to the data. No other normalisation was used.

Normalization template: No template was used for normalisation.

Noise and artifact removal: In vivo data were corrected for susceptibility and eddy current distortions using FSL topup and eddy. FSL melodic was used for ICA-based denoising of the resting-state data. Components were manually assigned as noise/signal based on characteristics in the spatial and frequency domain.

Volume censoring: Volumes were not censored.

Statistical modeling & inference

Model type and settings: Simple correlations were performed in MATLAB to relate ODI estimates from MRI and microscopy. The correlation coefficient r was calculated using MATLABs (2021a) fitlm function, and the p-value calculated using an F-test comparing the regression model to a degenerate model with only a constant term.

Effect(s) tested: No effects tested.

Specify type of analysis: ☐ Whole brain ☒ ROI-based ☐ Both

Anatomical location(s): Voxels were analysed from white matter masks in in vivo or postmortem diffusion MRI space. These masks were generated by first running FSLs Fast on the in vivo/postmortem structural for automatic white/grey matter segmentation. The postmortem white matter mask was then corrected by hand. These masks were subsequently mapped to diffusion space using appropriate FLIRT/FNIRT transforms. The centrum semiovale mask was drawn by hand.

Statistic type for inference (See Eklund et al. 2016): Simple correlations were performed voxelwise.

Correction: No correction was performed.

Models & analysis

n/a	Involved in the study
☒	☐ Functional and/or effective connectivity
☒	☐ Graph analysis
☒	☐ Multivariate modeling or predictive analysis
